# The university mathematics lecture: to record, or not to record, that is the question

**DOI:** 10.1007/s13394-023-00444-2

**Published:** 2023-03-07

**Authors:** Maria Meehan, Emma Howard

**Affiliations:** School of Mathematics and Statistics, University College Dublin, Dublin, Ireland

**Keywords:** Undergraduate mathematics, Face-to-face lecture, Online lecture, Lecture recordings

## Abstract

While recordings of lectures proved invaluable for students’ learning during the pandemic, as our university transitioned back to in-person teaching there was a call from many lecturers to remove the requirement to provide lecture recordings due to the perceived negative impact on attendance. To examine in detail the relationship between recordings and the corresponding face-to-face lectures, we conducted a study on the *formats* of lectures across our undergraduate mathematics programmes pre- and post-lockdown in March 2020, and students’ perceptions of how beneficial they felt each was for their learning. In May 2020, 156 mathematics students completed a survey containing both quantitative and qualitative questions. Findings indicate that pre-pandemic almost 70% of the lecture formats classified by students were traditional in nature, with 20% classified as containing some interactions, and the remainder as including group work. While students did not perceive *great* differences in terms of the benefits to learning between the face-to-face and online lecture formats in the majority of modules, those who experienced interactions or group work in lectures before lockdown, reported a greater drop than those who experienced a traditional lecture. Irrespective of preferences for face-to-face or online learning, students were clear about the value of lecture recordings and interactions with peers and lecturers to their learning. Based on our findings, and those of others, we discuss implications for mathematics lecturers’ practice. Specifically, we argue for the provision of lecture recordings or short pre-recordings, especially when the lecture is traditional in nature.

## Introduction

Our School of Mathematics and Statistics is situated in a large research-intensive university in Ireland. As we transitioned back to in-person lectures in the academic year 2021–2022, our university administration recommended that, where reasonable, recordings of all lectures should be made available to students to accommodate absences due to the virus still being in circulation. To facilitate this, most classrooms and lecture halls were fitted with lecture-capture technology. Anecdotally attendance at lectures (not just mathematics) has remained stubbornly low even as incidents of transmission of the virus have decreased. There may be several reasons for this. For example, students may not have acquired accommodation near campus on the off-chance that the pandemic would force learning online again, as it had the previous academic year. Or students, especially those who entered university in September 2020, may feel that they know only one way to learn at university—remotely. Others may have tried to maintain the flexible work-life-study balance they had developed over the lockdowns, which was incompatible with now attending all lectures in person. However, anecdotally lecturers from across a variety of disciplines are laying the blame firmly at the feet of lecture recordings. Their focus is on getting students back to the lecture and they believe that if recordings were not available, then this would be achieved. Speaking with colleagues in other universities we hear that similar conversations are taking place in their institutions. Therefore, with a focus on university mathematics, we feel it is timely to address the concerns of lecturers in relation to the provision of recordings. We attempt to do so in this paper by presenting findings from a study conducted in May 2020 with undergraduate students from our school, complemented with findings from the studies in the recent review by Lindsay and Evans (2021), along with others conducted on mathematics students’ perceptions of learning during lockdown (Hyland & O’Shea, 2021; Radmehr & Goodchild, 2022). It is our hope that this will permit university mathematics lecturers to make an *informed decision* about *whether* and *how* they could use lecture capture or recordings in their modules in the future.

The study presented in this paper had two aims when conducted in May 2020. One aim was a consequence of the first author’s role as head of teaching and learning in the school before, and during, the pandemic. As a return to ‘normal’ on-campus learning in the academic year 2020–2021 was looking increasingly unlikely at that time, we wanted to collect and analyse feedback from students on our mathematics programmes about their experiences of learning online in the first wave of the pandemic, in order to inform colleagues’ decisions about how to proceed with their teaching the subsequent year. To this end, an online survey was emailed to just under 900 students enrolled to modules in the School of Mathematics and Statistics, to which a total of 156 responded. Due to the urgency in providing colleagues with recommendations, responses to the open questions in the survey were analysed and in August 2020 a report was circulated to lecturers in the school with key findings from the analysis and recommendations for practice relating to the online delivery of modules (Meehan & Howard, 2020).

A second aim for the study stemmed from our research interests. Findings from our previous research (Howard et al., 2018; Meehan & McCallig, 2019) and that of others (Inglis et al., 2011; Le et al., 2010) on the provision of online videos (in the form of lecture capture or short, pre-recorded videos) to complement, and/or substitute for, the face-to-face lecture, show that when undergraduates are given the choice to attend lectures and/or watch online videos in mathematics modules, most tend to favour one over the other with different reasons for their decisions. In the spring trimester of 2020 students had no choice but to experience face-to-face lectures in the first 7 weeks of the trimester, and then experience completely online delivery in the remaining 5 weeks, albeit under very unusual circumstances. In almost all the modules the underlying subject and lecturer remained the same. We felt that this unique situation meant students could comment on how beneficial for their learning they found *both* the face-to-face and online lectures in the same underlying module with the same lecturer, whereas previously they may only have experienced, or engaged in, one. Moreover, we felt it was important to have a measure of the level of interactivity in lectures experienced by students—what we are calling the *format* of the lecture—for two reasons. The first reason was to explore whether students perceived a recording to be a good substitute for a more traditional face-to-face lecture, and less so for a more interactive face-to-face lecture. The second reason was to capture a picture of the format of face-to-face lectures in undergraduate mathematics modules in a fairly typical university pre-pandemic. We felt that such a baseline might prove important in the future if the pandemic were to cause a fundamental change in the teaching of undergraduate mathematics.

To address the second aim, we proposed the following research questions:

- (RQ1) What lecture formats did mathematics students experience in their modules in spring trimester 2019–2020 before the closure (BC) of in-person activities due to the COVID-19 pandemic, and how beneficial for their learning did they perceive them to be?
- (RQ2) What lecture formats did mathematics students experience in their modules in spring trimester 2019–2020 after the closure (AC) of in-person activities due to the COVID-19 pandemic, and how beneficial for their learning did they perceive them to be?
- (RQ3) How did the lecture formats and their perceived benefits to learning compare before the closure and after the closure of in-person activities?

We use the classification of lectures described in the study by Danielson et al. (2014) to describe the formats of the face-to-face lectures. They classified lectures as being either straight, interactive, or mixed with group work, increasing in levels of interactivity from the straight or traditional lecture, to interactive which features occasional interaction such as whole-class questioning, to mixed with group work. We use an adapted and extended version of the Danielson et al. classification to describe the format of the online lectures. Of the 156 students who completed the survey, 121 provided a total of 480 classifications and ratings covering approximately 50 undergraduate mathematics modules. We present the findings in this paper.

We will also highlight two main findings from the research questions that were addressed in the report to colleagues in August 2020 (the first aim of the study):

- (RQ4) What particular aspects (if any) of the online lecture formats that the mathematics students experienced after the closure of in-person activities did they perceive as beneficial for their learning, and why?
- (RQ5) Having experienced both in-person and online environments in spring trimester 2019–2020, what would mathematics students’ ideal blended learning environment for a mathematics module look like, and why?

We use the findings presented in this paper, along with those of others from the literature, to discuss implications for mathematics lecturers’ practice in relation to the decision about whether, and how, they might use lecture capture or recordings in their undergraduate modules.

We make a note about the use of the phrase ‘face-to-face’ throughout the paper, which in designing the survey, we used to represent in-person, on-campus lectures. Of course, one can legitimately have a face-to-face interaction with another person online too. However, since ‘face-to-face’ was the term used in the survey, and by students in their responses to the survey, we will use this term in reporting our findings on in-person, on-campus lectures.

## Literature review

In this review, we examine the literature on the use of lecture recordings in university mathematics. Given the backdrop to our study, we conclude by presenting some studies that examine undergraduate mathematics students’ perceptions of the move to online teaching at the start of the pandemic.

### Use of lecture recordings in university mathematics

Lindsay and Evans (2021) recently conducted a systematic literature review on the use of lecture capture specifically in the area of university mathematics. In this review they highlight that research into the use of lecture capture in university mathematics is not keeping up with the speed of adoption. Their paper was submitted a few months into the pandemic in July 2020, and they predicted that the lag would become even more pronounced post-pandemic given the explosion in online teaching and the development of video resources during various lockdowns that caused the sector to skip ‘a natural gradual stage of continuous development’ (p. 2). Sixteen studies, published between 2010 and 2019, were included in their review. They took lecture capture to encompass not just recordings of the live lectures, but also pre-recordings of content created by the lecturer, sometimes broken into 3–4 shorter recordings, but which corresponded to the content covered in a live lecture. (In this paper we will maintain the distinction between lecture capture (LC) and pre-recordings (PR), referring to them collectively as lecture recordings, or simply recordings.) The majority of studies in their review have as their focus one or two specific modules where recordings are used. Moreover, at least half the studies involve ‘service’ mathematics modules—modules tailored for students of Business, Engineering, or Physics—and the majority of the mathematics modules (‘service’ or otherwise) in the studies are introductory in nature. Finally, at least three pairs of studies in the review are examining the same modules in the same universities. From their review, we can conclude that there are in-depth studies into the use of recordings in a very small number of ‘service’ mathematics and introductory mathematics modules.

In their analysis of the articles, Lindsay and Evans (2021) found that of the seven articles examining the impact of the availability of recordings on attendance, three reported no change, and four reported a negative effect. In relation to student attainment, eight of ten articles reported a negative impact of recordings on it, and one each reported no impact or a positive one. They suggest that one reason why the availability of recordings might have a negative impact on attainment is that while it may be a ‘functional substitute for a highly motivated student’ (p. 13), it may permit ‘a less motivated student to get the impression of learning even when they are not cognitively engaged’ (p. 13). They point to a study by Trenholm et al. (2018) that suggests that students who do not attend lectures and rely on lecture recordings may result in them taking a surface approach to their learning. Another study by Yoon and Sneddon (2011) found that some students missed lectures with the intention of watching the recordings but never got around to it. In their discussion Lindsay and Evans go so far as to suggest that the decision to provide lecture capture raises a ‘potential ethical dilemma’ (p. 17) for universities—it can benefit some students, while possibly disadvantaging those who would come to lectures if recordings were not available. This underlines that mathematics lecturers are right to have the concerns and questions about lecture recordings, and there is not a straight-forward right-or-wrong response.

From the perspective of mathematics students, however, studies show that they place a high value on recordings (Lindsay & Evans, 2021). As noted earlier, when offered the choice to attend lectures or watch recordings, students generally have a preference (Howard et al., 2018; Meehan & McCallig, 2019; Inglis et al., 2011; Le et al., 2010). Students who prefer to watch recordings generally appreciate the flexibility they provide to study at a time that suits them, to avoid long commutes, or to study at their own pace (either faster or slower than in the lecture), while those who prefer attending lectures generally appreciate interactions that occur between students and between student and lecturer (such as the ability to ask questions), and the structure that scheduled lectures to provide (Howard et al., 2018; Hall et al., 2022; Wood et al., 2021; Yoon & Sneddon, 2011; Yoon et al., 2014). But even those students who generally prefer to attend lectures, describe how they use recordings to catch up on missed lectures; to review parts of a lecture that they found difficult; and to revise for examinations (Howard et al., 2018; Hall et al., 2022; Wood et al., 2021; Yoon & Sneddon, 2011; Yoon et al., 2014).

Lindsay and Evans (2021) focus on the issue of bringing students back to the live lecture and present two proposals. They suggest that lecturers may need to work on making lecture attendance more beneficial to students perhaps by ‘flipping’ the classroom. This means that instruction moves outside the lecture with students meeting new material perhaps through assigned readings or watching pre-recorded videos, and then the time in the lecture is spent engaging in activities based on this material. The second suggestion they give is to make lectures more interactive, noting that some of the articles included in the review reported that students appreciated opportunities to interact with other students and the lecturer.

For example, two of the articles included in the review by Lindsay and Evans (2021) were based on a study conducted at the University of Edinburgh, with a third article published more recently (Wood et al., 2021). In this article, findings from interviews with students who took at least one of three first-year courses in Mathematics or Physics are presented. Two of these courses used a flipped learning model while the other was more traditional in nature with the lecturer presenting material at a blackboard. Lecture-capture was available for all three courses. Students reported a preference for attending live lectures and felt there was an added value to attending lecture sessions where active learning took place. Recordings were used by students more often in the traditional course where time was spent on new content delivery resulting in a more information-dense session. The authors conclude that ‘if students feel attendance at live lectures has some discernible additional benefit, then they will attend them rather than watch online’ (p. 452).

While increasing the level of interactivity in a lecture may encourage more students to attend, it does raise the question about the utility of the lecture recording, specifically in the case where lecture-capture is used. Moving outside of university mathematics, Danielson et al. (2014) conducted a study at a College of Veterinary Medicine in the USA to explore, amongst other things, the relationship between the teaching approach taken in lectures and how useful students perceived the recordings of these live lectures to be. They classified lectures as being either straight, interactive, or mixed with group work, increasing in levels of interactivity from the straight lecture which is mainly a one-way presentation by the lecturer, to interactive which features occasional interaction using for example, whole-class questioning, through to mixed with group work which as the name suggests involves some instruction along with episodes of group work. They found an inverse relationship between how interactive the lecture was and how likely the student was to view the recording, with the student least likely to watch a recording of a lecture classified as mixed with group work. Consistent with studies in mathematics, students were overwhelmingly positive about the availability of lecture-capture and reported watching recordings to catch-up on missed lectures, to keep up with lectures that were fast-paced, to review parts of lectures, and to study for exams.

The proposal by Lindsay and Evans (2021) that lecturers make their lectures more interactive, coupled with the findings from the study by Danielson et al. (2014) and Wood et al. (2021), would suggest that in this instance lecture-capture acts more as a complement to the face-to-face lecture, rather than as a substitute. However, if one wants to cater to students’ preferences and offer both interactive face-to-face lectures and recordings, a suggestion might be to use short pre-recordings instead of lecture-capture. Meehan & McCallig (2019) describe how they designed a large service mathematics module where students were given the choice to attend (interactive) lectures and/or watch short pre-recordings. In order to help students navigate lectures and or recordings, fine-grained learning outcomes were stated and the lectures, short recordings, and problem sets were designed around these. Given the range of mathematical backgrounds of the large cohort of students, the purpose of the design was to allow students to take as little, or as much, time as they needed to master the material. Those with high prior achievement could efficiently cover content by watching short videos, while students with low prior achievement were encouraged to attend lectures and watch videos as necessary. A weekly quiz based on groups of learning outcomes ensured students kept up-to-date with content and provided feedback on mastery of topics. The authors note that this design may only be suitable for large, introductory, or service modules.

While a detailed discussion of the literature on the format of mathematics lectures and mathematicians’ beliefs about the best way to teach mathematics is beyond the scope of this paper, we conclude this section by mentioning two studies that we believe are relevant. The first is a recent survey of the literature on the teaching and learning of proof-based mathematics courses (Melhuish et al., 2022). The authors note that the predominant form of instruction in such courses is the traditional lecture, where mathematicians write the formal mathematics on the board while providing informal meta-commentary. Moreover, studies included in the review indicate that most mathematicians believe this is the best way to teach such courses and can provide strong reasons for taking this approach. Given that this mode of instruction aligns with mathematicians’ beliefs and goals about teaching and learning mathematics, changing their practice may be difficult.

The second study we mention is by Meehan (2019). In it she reflects on what she noticed while teaching a first-year mathematics course where students had the choice to attend lectures and/or watch recordings. In terms of changes to practice, Meehan describes how she struggled not to fall into an old pattern of complaining that some students were not attending lectures, as she would have done before recordings were introduced. However, on the positive side, she noticed better engagement on in-class activities amongst those students who did attend lectures and realised that recordings had the potential to make students more responsible for their learning.

### University mathematics education during a pandemic

The mathematics education research community has been proactive in capturing students, teachers, and lecturers’ experiences and perceptions of the sudden move to online teaching in Spring 2020. For example, special issues of Educational Studies in Mathematics (Chan et al., 2021), the International Journal for Mathematical Education in Science and Technology (IJMEST) (Seaton et al., 2022), and Teaching Mathematics and its Applications (Gillard et al., 2021) have been dedicated to the topic.

Two studies that focused specifically on undergraduate mathematics students’ perceptions and experiences of the move to online teaching in Spring 2020, were conducted by Hyland and O’Shea (2021) and Radmehr and Goodchild (2022). These studies found that undergraduate mathematics students reported that the loss of student–student and student–lecturer interactions impacted them negatively after the move to online delivery. Students reported feeling isolated, anxious about their learning, and lacking in motivation (Hyland & O’Shea, 2021). When highlighting what they would like to see happen in the subsequent academic year, students viewed interactions with lecturers, tutors, and other students as essential. Challenges associated with the loss of these interactions, such as feelings of isolation, missing peers to work with, along with not being able to ask questions of lecturers and tutors in person, were also experienced (Radmehr & Goodchild, 2022). However, some students cited benefits to studying at home: money and time saved on commuting; the ability to work at their own pace; and, access to additional resources (Hyland & O’Shea, 2021).

## Method

### Participants

The School of Mathematics and Statistics in University College Dublin offers eleven undergraduate degree programmes, under four types of degree programmes. In May 2020, an electronic survey designed to address the five research questions was emailed to just under 900 students who were enrolled to these undergraduate degree programmes. In total, 156 responses were received (75 male, 79 female, 1 non-binary, and 1 preferred not to say), and the breakdown of respondents is provided in Table 1. While the overall response rate is low at approximately 17%, the response rate for Stages 2 and 4 of the Bachelor of Actuarial and Financial Studies and Stages 3 and 4 of the Bachelor of Science programmes is relatively high. For the study, we will refer to all students as ‘mathematics students’.

**Table 1:** Number of respondents from each stage of main degree programmes

Degree programme	Stage 1	Stage 2	Stage 3	Stage 4	-	Total
Bachelor of Actuarial and Financial Studies (BAFS)	9 (16.1%)	19 (38%)	*P*	12 (23.5%)		40
Bachelor of Science (SCI)	21	21	18 (20.5%)	28 (33.7%)		88
Bachelor of Arts (BA) (Joint Major)	5	1	6	NA		12
BSc Social Science (SS) (Joint Major)	8	7	NA	NA		15
Undeclared		1				1
Total	43	49	22	40	2	156

*P* indicates students were on work placement for the spring trimester

### Data collection

The anonymous online survey was operationalised using Qualtrics software. In the first part of four, students were asked for demographic data. The second part was aimed at addressing RQ1-RQ4. Modules in the School of Mathematics and Statistics carry one of four subject code types: ACM for Applied and Computational Mathematics; MATH for Mathematics; MST for Mathematical Studies; and STAT for Statistics. As we did not want specific modules identified for reasons of ethics, we requested students to give pseudonyms to each of the *n* modules they were enrolled to as follows: assign a module as Module 1 on the survey. From a drop-down menu, choose whether this module is ACM, MATH, MST, or STAT, and from another drop-down menu choose the level of the module, for example, 1, 2, 3, or 4. Repeat this for each module that you are enrolled in. In this way, we captured the discipline and level associated with the module, but not the identifying code or title.

To assist students in categorising the face-to-face lecture format of modules BC, we provided the description of the three teaching approaches in lectures from Danielson et al., (2014, p. 123) in the survey as follows:

#### Face-to-face lectures

The format of face-to-face lectures can be categorised as one of three types: straight lecture, interactive lecture, or mixed lecture and group work.

- **Straight lecture:** features almost exclusively formal in-class presentation by the lecturer.
- **Interactive lecture:** features occasional interaction, such as answering clicker questions or whole-class questions, in a predominantly lecture-based context.
- **Mixed lecture and group work:** features a combination of lecture accompanied by group work.

For each module, students chose one of these three lecture formats from a drop-down menu and then rated how beneficial they perceived this type of lecture format to be for their learning from 1 *not at all beneficial* to 5 *very beneficial*.

In relation to categorising the online lecture format in modules AC, the first author was aware of a range of options that lecturers were providing. Some were providing live online lectures with recordings available later, therefore in keeping with the Danielson et al. (2014) categorisation, we presented three categories of live lectures: *live straight lecture*; *live interactive lecture*; and *live mixed lecture with group work*. Other lecturers were pre-recording lectures (either whole lectures or short videos with 3–4 per lecture) and therefore, we also had the categories of *pre-recorded video lecture*, and *pre-recorded short videos*. Some staff members were making podcasts to accompany notes, others were recommending existing online videos, and some were providing notes only. Therefore the following was provided on the survey:

#### Online lectures

The format of online lectures can be categorised into one of eight types.

- **Pre-recorded video lecture by lecturer:** lecturer pre-records full lecture and makes it available online.
- **Pre-recorded short videos by lecturer:** lecturer pre-records series of short videos, e.g. 5–15 min, rather full lecture.
- **Videos recommended by the lecturer:** lecturer recommends YouTube videos, or similar.
- **Live straight lecture with recording made available later:** lecturer recommends YouTube videos, or similar.
- **Live interactive lecture with recording made available later:** features straight lecture delivered live at allocated time by lecturer using Virtual Classroom, Skype, zoom, or similar, with recording of lecture made available online afterwards.
- **Live mixed lecture and group work with recording made available later:** features mixed lecture and group work delivered live at allocated time by lecturer using Virtual Classroom, Skype, zoom, or similar, with recording of lecture made available online afterwards.
- **Podcasts:** lecturer pre-records podcasts.
- **Notes only:** lecturer provides own notes with no accompanying videos or podcasts.

As with the face-to-face lecture format, for each module students chose one of these formats from a drop-down menu and then rated how beneficial they perceived this type of format to be from 1 *not at all beneficial* to 5 *very beneficial*. The second part of the survey concluded with the following open-response question, which aimed to address RQ4: what particular aspects (if any) of the online lecture formats that you experienced were beneficial to your learning, and why?

The third part of the survey was similar to the second part except that its focus was on tutorials and it included the open-response question: what particular aspects (if any) of the online lecture formats that you experienced were beneficial to your learning, and why? We will not discuss the quantitative data gathered on tutorials in this paper. And finally, the fourth part, aimed at addressing RQ5, consisted of the following open-response questions:

Blended learning involves lectures, tutorials, and assessments being delivered via a combination of online and face-to-face formats. Having now experienced both face-to-face and online environments, what would your ideal blended environment for ACM/MATH/MST/STAT module look like, and why? (Where possible, make reference to lectures, tutorials, and assessments in your answer.)

### Data analysis

While the survey was anonymous, each student was given an unique identifier based on their programme (BA, BAFS, SCI, or SS—see Table 1), stage (1–4), and gender (M or F). The second author analysed the quantitative data graphically and used descriptive statistics. Ratings were analysed based on students’ different stage, programme, and gender, as well as the for the lecture format classification (BC and AC).

The responses to the three open-response questions along with student identifiers were exported to the software package *NVivo* for analysis. The six phases of thematic analysis outlined in Braun and Clarke (2006) were followed. The first author familiarised herself with the data. She then performed two rounds of coding using an open coding approach. In the first round, responses to the two questions on what students found beneficial about the online lectures and tutorials were analysed separately to the responses about students’ preferred blended learning environments. A second round of coding was then performed on the entire data set. She then sorted the codes into potential themes, ten in total. No theoretical framework was used in analysing the data. However, as the purpose of the analysis was to determine what students found useful or not, and why, the first author approached the data with this in mind, and several of the codes reflect this. For example, one theme is *OL Lecture* which contains the codes *OL Lecture Beneficial* and *OL Lecture Not Beneficial*, which each contain seven subcodes. In the fourth phase, as well as reviewing the themes, she felt that one of the themes ‘Why recommend?’ required further analysis. In this theme she had collated codes that related to the reasons why students made a particular recommendation, or had a particular preference, irrespective of what that recommendation or preference was. She went back over the entire data set to ensure that all relevant data was coded to this theme. The themes were then named and defined. An internal report outlining findings on students’ perceptions on the affordances and constraints of the online learning environment circulated to staff in August 2020 (Meehan & Howard, 2020).

## Results

Of the 156 students who completed the survey, Table 2 shows the number who classified and rated both the face-to-face and online lecture formats of *n* modules, where *n* = *1,…,7*. This accounts for 121 students and 480 module classifications and ratings. The remaining 35 students only partially classified and rated their modules. To avoid confusion, we will present the results for the 480 modules with complete classifications and ratings. The reader should note that these are not 480 distinct modules—rather 480 classifications of *N* modules. As we did not gather data on specific module codes and titles, we do not know the number of distinct modules, *N*, although based on programme and stage demographics we estimate that the total number of unique modules is at least 50. Finally, we note that all but two students answered some or all of the three open-response questions, and generally students gave quite detailed responses, particularly those from Stages 3 and 4.

**Table 2:** Number of students who classified and rated the face-to-face and online lecture formats of n modules, where *n* = 1,…,7 and corresponding number of module format classifications and ratings

No. modules classified and rated by student	No. students	Total no. module classifications and ratings
1	7	7
2	14	28
3	23	69
4	30	120
5	28	140
6	17	102
7	2	14
Total	121	480

We now present the findings for each of the research questions, starting with RQ1-RQ3, and finishing with a selection of findings for RQ4-RQ5.

### RQ1. Face-to-face lecture format classifications and ratings

**Fig. 1:**
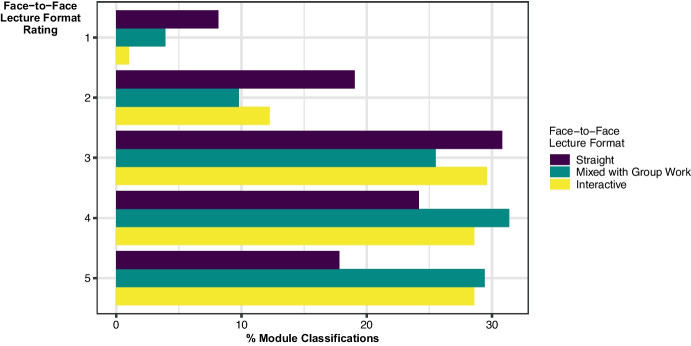
Percentage of module classifications for each face-to-face lecture format as perceived by students to benefit learning from 1 not at all beneficial to 5 very beneficial

In terms of the face-to-face lecture formats, of the 480 module classifications, 69% were *straight lecture*, 20% were *interactive lecture*, and 11% were *mixed lecture and group work*. When the distribution of lecture format classifications across the four subject types was examined—ACM, MATH, MST, and STAT—little difference was observed. Therefore, from now on we will refer to all modules as ‘mathematics modules’. There was also little difference observed across levels of modules, although Level 3 and 4 modules had a slightly higher percentage of *interactive lecture* and *mixed lecture and group work* format classification than the lower levels. The percentage ratings associated with these module classifications are given in Fig. 1. Students rated 61% of the module classification *mixed lecture and group work* as being either beneficial or very beneficial, to their learning, with the corresponding figures for *interactive lecture* and *straight lecture* at 57% and 42%, respectively. Given that the *straight lecture* format was the most predominant classification, it is noteworthy that students rated 31% of these as moderately beneficial to learning, with 19% rated as slightly beneficial, and 8% as not at all beneficial.

### RQ2. Online lecture classifications and ratings

**Fig. 2:**
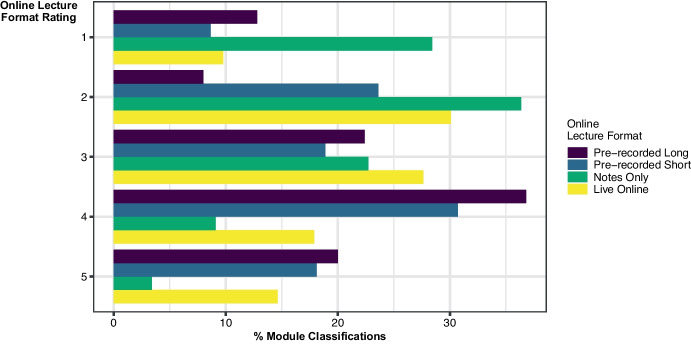
Percentage of module classifications for three most frequently occurring online lecture formats along with the three live formats combined as live online, as perceived by students to benefit learning from 1 not at all beneficial to 5 very beneficial

Of the 480 online lecture module classifications that were rated, *pre-recorded lecture* and *pre-recorded short video* were most prevalent with both at 26%, with *notes only* next at 18%. The three live formats (with recordings available online after) that mirrored the face-to-face lecture formats made up 26% of the online module classifications, with *live straight lecture* and *live interactive* accounting for the majority of these at 14% and 10% of the total respectively. The remaining 4% of module classifications were *podcasts* or *recommended video*. Combining the three live categories under the heading *live online,* the percentage ratings associated with this classification, along with the other three most frequent formats is given in Fig. 2. Perhaps unsurprisingly, students do not perceive that notes alone are beneficial for learning. *Pre-recorded lecture* format was the most frequent classification; therefore, it is noteworthy that students rated 20% of these as very beneficial to learning, with 34% rated as beneficial, and 24% as moderately beneficial. *Live online* did not fare as well, with just under 10% rated as not beneficial for learning, and 30% as slightly beneficial.

### RQ3. Face-to-face lectures to online lectures—classifications and ratings

**Fig. 3:**
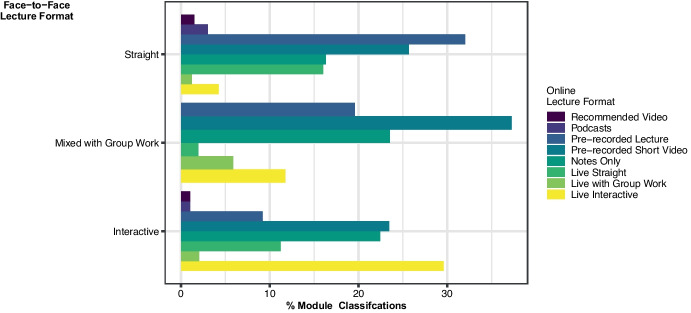
Percentage of each face-to-face lecture module classification that pivoted to each of eight online lecture formats

For each face-to-face lecture format, the percentage of module classifications that pivoted to each of the online lecture formats is presented in Fig. 3. Over 75% of each of the face-to-face lecture formats pivoted to either a pre-recorded format (lecture or short videos) or one of the three live online formats with recordings available later. Of the module classifications that had a *straight lecture* face-to-face format initially, 58% pivoted to a pre-recorded format and 21% to a live online format, with the percentages for interactive lecture being 32% and 43% respectively, and 57% and 20% respectively for *mixed lecture and group work*.

To explore whether there is a connection between how beneficial students perceived the face-to-face and online lecture formats of a given module to be, we let

1 $$d=\text{rating for face-to-face format }-\text{ rating for online lecture format.}$$

**Fig. 4:**
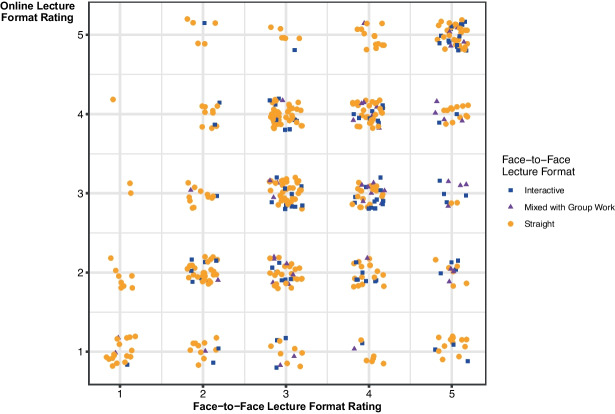
The face-to-face lecture rating and format for each module classification with corresponding rating for online lecture format

The *d*-values for each face-to-face format are provided in Table 3 and the ratings for each module format BC and AC are presented in Fig. 4. By way of illustration, consider the 12 points graphed at the coordinates (5, 2). This means that 12 modules were rated as being 5 when offered face-to-face, with the rating dropping to 2 when they moved online. In this instance *d* = 3. Furthermore, the circles, squares, and triangles denote which were originally offered as *straight lecture*, *interactive lecture*, and *mixed lecture with group work*, respectively.

**Table 3:** Number and percentage of each face-to-face lecture format classification with a given d-score, where d =  −4,…,4

Lecture Format	*d* = −4	*d* = −3	*d* = −2	*d* = −1	*d* = 0	*d* = 1	*d* = 2	*d* = 3	*d* = 4	% (and total number) for each format
Straight lecture	0% (0)	2% (6)	5% (17)	20% (66)	40% (133)	18% (61)	7% (24)	4% (12)	4% (12)	100% (331)
Interactive lecture	0% (0)	1% (1)	3% (3)	10% (10)	41% (40)	22% (22)	14% (14)	5% (5)	3% (3)	100% (98)
Mixed lecture and group work	0% (0)	0% (0)	0% (0)	8% (4)	35% (18)	35% (18)	14% (7)	8% (4)	0% (0)	100% (51)
% (and total number) for each *d*	0% (0)	1% (7)	4% (20)	17% (80)	40% (191)	21% (101)	9% (45)	4% (21)	3% (15)	100% (480)

Examining Fig. 4, we note that the face-to-face and online lecture formats of 191 module classifications (40%) have been rated the same by students (*d* = 0). The online lecture format rating for 107 module classifications (22%) increased from the corresponding face-to-face lecture rating (*d* < 0), with the rating of 80 module classifications (17%) increasing by one (*d* =  −1). On the other hand, the online lecture format rating for 182 module classifications (38%) decreased from the corresponding face-to-face lecture rating (*d* > 0), with the rating of 101 modules (21%) decreasing by one (*d* = 1). In summary, the online lecture format rating of 372 module classifications (78%) is within plus or minus one of the corresponding face-to-face lecture format rating, suggesting that in the pivot to online delivery, for a large proportion of the modules rated, students did not perceive the online format in a module to be *particularly* more or less beneficial to learning than they perceived the face-to-face lecture format of that module to be.

Examining each face-to-face lecture format separately though, we see that in the move to online delivery, the ratings of 18% of the *straight lecture* classifications decreased by one (34% overall), while 20% increased by one (27% overall). However, 22% of the ratings of the *interactive lecture* classifications decreased by one (44% overall), while only 10% increased by one (13% overall) in the move to online. The corresponding values for *mixed lecture and group work* are 35% decrease by one (57% overall) and 8% increase by one (with 8% overall also). Therefore, while 78% of module classifications are within plus or minus one of the corresponding face-to-face lecture format rating, students who experienced interactive lectures or lectures with group work embedded in them before lockdown, reported a greater drop in the perceived benefits to learning from the online formats, than those who initially experienced a traditional lecture.

Bearing in mind that they account for just 8% of the total module classifications, we examine the extremes in the top left-hand corner (*d* =  −3) and bottom right-hand corner (*d* = 3 or 4) of the graph in Fig. 4 in order to investigate if the corresponding modules have anything in common with regard to lecture formats. There are seven module classifications by seven distinct students with *d* =  −3. The face-to-face lecture format of six of the lectures was described as *straight lecture,* the other was *interactive lecture,* while in relation to the online lecture formats, one was live, five had pre-recorded materials, and one had notes only. To explore possible reasons for the ratings, we examine the qualitative responses of the students. The value of recordings to students’ learning was clear. Having a recording to pause and rewind when working through material, or revising, was beneficial, especially in more difficult modules.

For some of the more difficult modules with a lot of content, it is good to have the lecture videos or podcasts so I can pause and go over specific things. Whereas in a face-to-face lecture, I would fall behind and lose concentration (SCI3, M4).

Recordings enabled students to study the lecture material at their own pace, both review it when the lecturer went too fast, or to skip material that they already knew.

Having all notes and videos available lets me go back over any topics that I might have missed in a lecture. It also allows me to skip parts that I've seen many times before, saving me time (SCI4 M4).

The ability to take better-written notes using recordings was also noted. We highlight that the value of recordings to learning was cited by several students, irrespective of whether they explicitly stated a preference for online or face-to-face learning in their comments. However, two of these seven students indicated a preference for online delivery:

Majority of things can be done online. The only advantage with face to face lectures is being with your friends. Other than that, everything about online delivery is better (SCI4 M11).

I actually found that I learned things much better post-closure and I really hope that some of teaching methods carry over after [the university] reopens (SCI4 M4).

Examining some of the modules whose ratings lie in the bottom right-hand corner of Fig. 4, we observe that the face-to-face lecture format classifications of fifteen modules, by five distinct students, was perceived to be very beneficial for learning, yet the online lecture format was perceived to be not at all beneficial for learning (*d* = 4). In fact, two of these students rated all their modules in this way, accounting for eleven of the fifteen modules. Both students were clear in their overall comments that they did not like learning online, irrespective of the formats that the modules took.

I found it almost impossible to learn from the online videos. I honestly did not find anything about online lectures beneficial. […] It’s the learning from home, no interaction, 100% of lectures online that I am really struggling to deal with (BAFS2 F2).

The other student felt learning had ‘got a lot more difficult’ (BAFS2 F3). The remaining four students were more measured in their responses, indicating that it was the online format of specific modules that they did not find useful. For example, one student had *d* = 4 for two of her five modules where the lecture format of both had moved to *notes only*. This is what perplexed her as she felt that ‘it is extremely difficult to teach mathematics to yourself with little outside influence’, but she did note that ‘in cases where full pre-recorded video lectures are available, this is perfectly acceptable’ (SCI3 F5). Another student, similarly rated one of her modules that switched to *notes only*. The two remaining students were generally positive about online lectures, with one noting the benefits for students who had to commute, and the other proposing a move to ‘flipped’ learning post-pandemic.

Recorded lectures/zoom tutorials being standard could really help someone who would otherwise spend 4 h (2 each way) in a bus to reach the one or two lectures they have on that day (SCI4 M2).

A further twenty-one module classifications were given a *d*-rating of three by nineteen distinct students. Thirteen of these online module classifications were *notes only* and two had *videos recommended by the lecturer*. Some of these students do seem to be quite negative in their comments towards online learning. For other students in this cohort, rating one module’s online format as not beneficial for learning did not mean that they disliked online learning—the fact that three of these students have module classifications and ratings in the top left-hand corner of Fig. 4 (*d* =  −3) is testament to this.

### RQ4. and RQ5. Interactions and the availability of lecture recordings

In responding to the open-ended questions students gave many reasons why they liked one resource over another, and/or provided positives and negatives for a particular resource. Some of these are simply student preferences—one likes live online lectures because they know they cannot pause them, another likes pre-recorded because they can; one finds it difficult to ask questions in a face-to-face lecture, another finds it more difficult online. However, some aspects of online and face-to-face learning seem to transcend individual choice and are valued by all students. The two main aspects we found are firstly, the value that students place on the availability of recordings, and secondly, the value that students place on interactions with peers and lecturers. (These codes along with their subcodes and the number of references resulting from the qualitative analysis can be found in the Appendix (Table 4)).

First, irrespective of whether students expressed a preference for mainly online or face-to-face learning, a large cohort felt that recordings of lectures should be made available. They talked about using recordings to catch up on missed lectures, but also to review parts of a lecture they had attended but not fully understood, and to revise for examinations. The flexibility that recordings afford for students to engage with the material in their own time is appreciated, especially for those with caring responsibilities or with a long commute. Students appreciated the ability to study and engage with material at their own pace, often pausing and rewinding recordings. Others felt that notes written during this process were more beneficial to them than those taken in lectures. For others, recordings were a more efficient way to study, as they could skim over the content they knew already.

Second, the importance of interactions—both student–student and student–lecturer/tutors—were highlighted frequently both by students who had a preference for face-to-face learning and also those who were generally positive about online learning. In describing aspects of the online environment that students felt were beneficial for their learning, many involved opportunities for students to interact with each other or lecturers. Examples are online, break-out rooms; encouraging the use of the chat function during online sessions; online discussion forums; and, zoom office hours. Also in describing what their ideal blended learning environment might look like, interactions with friends and lecturers featured prominently. Having someone that you knew you could approach if you had a question was highlighted as important. As one student noted, it is ‘more difficult to discuss mathematical problems and questions through text/video’ (BAFS1 F7), and for this reason, perhaps mathematics students felt the absence of face-to-face discussion with peers and lecturers quite keenly. Indeed for some, it was its absence that made them realise the role it played in their learning.

However, I really missed the interactions with my peers and the collaborative element to learning. It has made me realise how much I learn from the people around me and everyone being based on campus provides a great environment for collaborative learning (SCI2, F3).

Easy accessibility to lecturers and tutors was also missed. Posting a question on a discussion forum or emailing the lecturer was not considered by some to be as effective as just speaking with them after class. In describing their ideal blended learning environment several students described a ‘flipped’ model where notes or videos are posted in advance of a live session, which would then involve students working on problems and interacting with each other and the lecturer:

I think nothing was lost by having face-to-face lectures prerecorded online. There is very little engagement in person at any of my lectures and I think people were more inclined to ask questions online. It is easier to understand the material when you can pause and understand it as it is explained to you. […] Obviously, it is difficult with funding, etc. to do a lot differently but I think ideally face to face time would be much more interactive to deepen understanding and applications of material with prerecorded content used to explain content initially (BAFS2 F1).

## Discussion

Pre-pandemic, the *straight lecture* (69%) was the most predominant face-to-face lecture format as classified by the undergraduate mathematics students in our study, followed by an *interactive lecture* (20%) and *mixed lecture with group work* (11%). In terms of how students rated these formats as benefiting their learning in modules, *mixed lecture with groupwork* had the highest percentage of classifications rated as beneficial or very beneficial for learning at 61%, followed by an *interactive lecture* at 57% and *straight lecture* at 42%. We found no difference in the spread of lecture formats amongst the four areas of concentration: ACM, MATH, MST, and STAT. While it is important to consider the impact of the underlying discipline when conducting research such as this (Lindsay & Evans, 2021), the sub-discipline level may be too granular to expect to see differences.

It is unsurprising that the predominant face-to-face lecture classification was *straight lecture* given its central pedagogical role in the history of the university (Friesen, 2011). In mathematics, the literature suggests it is the predominant mode of instruction in proof-based courses (Melhuish et al., 2022). Given that 42% of the *straight-lecture* classifications were perceived by students as being beneficial or very beneficial to learning is a reminder that it is what happens in the traditional lecture, how the students engage with it, and indeed how it sits in relation to other components of the module, that matters.

The module classification *mixed lecture with group work* format is rated more highly by students in terms of benefits to learning. Although we did not ask students to justify their ratings, the obvious explanation may be that students appreciate the active aspect to their learning, and this aligns with qualitative responses focusing on the value of interactions. While this format can theoretically be used with large groups, in practical terms it is more feasible when enrolment to a module is relatively low. Therefore it is unsurprising that Level 3 and 4 modules which generally have lower enrolments, had slightly higher proportions of *interactive lecture* and *mixed lecture with group work* formats.

In addressing RQ2, we see a high number of pre-recorded lectures and short videos in the module classifications. One explanation is that our school offers an online graduate programme, and some staff who have expertise in producing videos gave a workshop to others in March 2020. Anecdotally, more lecturers gave live online lectures the following academic year. This may be explained by lecturers having more familiarity with the relevant digital platforms and/or perceiving that pre-recordings take more time to make than giving a live lecture. In addition, the live lecture is closer to their usual face-to-face practice.

In relation to RQ3, findings suggest that the change in lecture format from BC to AC did not result in a *significant* increase (*d* <  −1) or decrease (*d* > 1) in terms of perceived benefits to learning for 78% of the module classifications. Therefore if the face-to-face format of a module was rated as being very beneficial to learning BC, its online counterpart was pretty similarly rated. The same is true if it was rated as not at all beneficial to learning. As one student noted:

- Whether live or online, quality of teaching usually came down to organisation, quality materials, going through topics in depth. Therefore classes which were good pre-lockdown tended to also be good post-lockdown. (BAFS4 M5).

Of the modules initially classified as having a *straight lecture format*, 40% of the ratings remained the same, while 20% increased by one. Over three-quarters of the straight lecture classifications pivoted to either a pre-recorded format or a live, online format with recordings available later. This suggests that students perceived these formats to be pretty good substitutes for the traditional lecture. This echoes the findings of Danielson et al. (2014) and Wood et al. (2021). Students’ perceptions of the benefits to learning of the online formats to the *interactive lecture* and *mixed lecture with group work* were less positive, with the largest drop in ratings for the latter format (57%). Maintaining active learning practices was a challenge for lecturers in the switch to online teaching. For example, Webb (2021) describes how a Networked Improvement Community of 23 universities in the USA who were involved in implementing active learning practices in their Calculus courses pre-pandemic, grappled with trying to sustain these practices during the lockdown.

When the qualitative comments were matched with the students who rated the online format of the lecture classifications as being significantly more beneficial to learning than the face-to-face formats, we identified some students who seemed to favour the online environment more generally for the flexibility and additional support it provided. On the opposite end, examining the students’ qualitative comments we see roughly two types of students—those who see no benefit to online learning more generally, and those who are just critical of how one or two modules were delivered online. However, it is also important to note that the extremes discussed here account for just 8% of the total module lecture format classifications.

The call by students for the provision of lecture recordings irrespective of whether lectures are online or face-to-face comes as no surprise, as the value students place on them is well-documented (Lindsay & Evans, 2021). The reasons students provide for how they use them are consistent with findings from other studies on mathematics students’ use of lecture recordings (Howard et al., 2018; Hall et al., 2022; Wood et al., 2021; Yoon & Sneddon, 2011; Yoon et al., 2014). Several students highlighted the role that recordings play in students’ note-taking, with Wood et al. (2021) reporting similar findings. This in turn indicates the value students place on having a good set of written notes to study, and we question whether this is something that may be specific to mathematics. Finally, in this study and others (Hyland & O’Shea, 2021; Radmehr & Goodchild, 2022) we saw that learning in lockdown brought to the fore the importance of interactions with peers and lecturers in supporting and motivating students’ learning, both inside and outside the classroom.

A limitation of this study is its self-report nature. We asked students to rate how beneficial they found various resources and in examining some of the extreme ratings above we saw that, for a small number of students, their predispositions for face-to-face teaching and learning may have impacted their subjectivity. We do not have codes or titles for the modules classified in the study, therefore we cannot say for certain how many are included, although the estimate is at least fifty. It could also be argued that students’ views about their online experiences during a pandemic, may give us very little indication of how they might perceive and use the online resources in more ‘normal’ times. And given most lecturers’ inexperience with online delivery, it may also have been unfair to ask students to rate their efforts at online teaching in an ‘emergency’ situation. However, if students found an online resource useful, missed a face-to-face resource, or cited a desire to see some digital resource embedded into future teaching, then we believe there is value to this in considering our future practice.

### Implications for practice

To frame the remainder of our discussion we return to the issue of addressing mathematics lecturers’ concerns about the availability of lecture recordings and the impact of recordings on lecture attendance. In this paper, we have highlighted the importance of the format that the lecture takes when considering this issue. Therefore we first consider responses to lecturers who provide lectures that are mainly traditional in format.

#### Should I provide recordings of my (traditional) lecture?

We strongly recommend the provision of recordings in this situation. Our findings and others (Danielson et al., 2014; Wood et al., 2021) suggest that students perceive recordings to be good substitutes for the traditional lecture. In addition to those students who use recordings as a substitute for the lecture, our findings and others (Howard et al., 2018; Yoon et al., 2014) highlight the many ways that students use recordings to complement their learning, even when they have attended the lecture. We argue against removing a resource that research has shown to be a beneficial study aid for students.

#### Will attendance at my (traditional) lectures drop if I provide recordings?

While findings from research on the impact of recordings on attendance are mixed (Lindsay & Evans, 2021), findings from this study and that of others (Howard et al., 2018; Inglis et al., 2011; Le et al., 2010; Meehan & McCallig, 2019) suggest that when given the choice between attending lectures or watching recordings, students generally have a preference. Therefore it is likely that some students will watch recordings instead of attending lectures because quite simply they prefer them, or because they offer the student the flexibility to learn at a time that suits them. In this instance, the lecturer may need to reflect on how they view poor attendance. By accessing engagement analytics on the university’s virtual learning environment, the lecturer can get a broader view of the number of students interacting with the module. In addition, it is worth remembering that just because a student attends a lecture, it does not mean they are learning, and indeed attendance without engagement may also give the students the ‘impression of learning’ (Lindsay & Evans, 2021, p. 13). Nonetheless, one should not underestimate just how difficult it is to adjust how one views and reacts to non-attendance in the traditional lecture (Meehan, 2019). We argue that it requires a change in mindset on behalf of the lecturer.

#### How will I know my students are learning if they are not attending the traditional lectures?

Of course, the terse response here could be: ‘How do you know they are learning when they attend the traditional lecture?’ Admittedly, when delivering a traditional lecture, lecturers may be monitoring non-verbal feedback from students and adapting their practice accordingly (Meehan et al., 2017). It is likely that lecturers value this feedback mechanism. However, there are other ways to gather feedback on learning such as monitoring questions on discussion boards, feedback from tutorials, correcting homework, or analysing weekly quiz scores. Perhaps ironically, reduced numbers in a large lecture may actually facilitate the lecturer having more quality interactions with those who do regularly attend (Meehan, 2019).

Lecturers may also be concerned that students will miss lectures with the intention of watching the recordings but never get around to it (Yoon & Sneddon, 2011). Or that in trying to watch several recordings in rapid succession (like binging on a box-set) prior to an assessment, they end up taking a surface approach to learning (Trenholm et al., 2018). In this case, short frequent quizzes, providing summative or formative feedback, could incentivise students to keep up with the material, if this is appropriate for the content of the module (Howard et al., 2018; Meehan & McCallig, 2019), while also providing essential feedback to lecturers.

#### My lectures have an interactive format—should I provide recordings?

Studies have shown that students find less value in recordings of interactive lectures (Danielson et al., 2014; Wood et al., 2021). Where students are expected to interact with the lecturer and/or peers in a lecture, then we suggest that lecture-capture is not a substitute for the lecture. Students may also have concerns about interacting with the lecturer when these interactions are being recorded and made available for the class. In this instance, the lecturer may want to make short pre-recordings of any exposition that will be included in the lecture (Meehan & McCallig, 2019). Or if the interactive lecture is captured as a whole, then it could be spliced and only the sections including exposition provided to the students.

#### If I make my lecture more interactive, will attendance increase?

Research has shown that students who attend lectures value interactions (Howard et al., 2018; Wood et al., 2021; Yoon et al., 2014), and for this reason, Lindsay and Evans (2021) recommend making lectures more interactive or flipping the lecture in order to encourage students back to the lecture. And indeed, this study and others conducted during the pandemic (Hyland & O’Shea, 2021; Radmehr & Goodchild, 2022), illustrates the value that students place on student–lecturer and peer-to-peer interactions. Whether more interactive lectures will result in an increase in attendance is unknown, especially in the wake of the pandemic. While many students missed interactions during lockdowns, they also experienced the flexibility that comes with learning online.

## Conclusion

Whether, and in what ways, the university mathematics landscape will change after the pandemic is yet to be seen. The traditional university lecture has a long history and has survived many technological innovations. We believe it is very unwise to turn our backs on resources such as recordings when research shows that students view them as good substitutes for the traditional lecture and that all students, irrespective of their preference for face-to-face or online learning, use recordings to support their learning. We argue that the focus must move to how they can enhance student learning. Education researchers, especially those in mathematics departments, must engage with these issues as lecturers’ practice must be informed by evidenced-based research, and not gut reactions.

## Appendix

**Table 4:** Name of subcodes and number of references of each for the codes interaction and use of recordings

**Name of code (bold) and subcodes**	**No. of references**	**Name of code (bold) and subcodes**	**No. of references**
**Interaction**	-	**Use of recordings**	-
Interaction general	10	Catch up	11
Ability to ask questions	47	Doing problems	1
Interaction with lecturer	32	Efficiency	8
Interactions with students—Learning	16	Flexibility	16
Interactions with students—social	5	Make notes	16
		Own pace	16
		Pause and rewind	36
		Review	32
		Revision	20
